# Hedgehog-mediated regulation of PPARγ controls metabolic patterns in neural precursors and shh-driven medulloblastoma

**DOI:** 10.1007/s00401-012-0968-6

**Published:** 2012-03-11

**Authors:** Bobby Bhatia, Chad R. Potts, Cemile Guldal, SunPhil Choi, Andrey Korshunov, Stefan Pfister, Anna M. Kenney, Zaher A. Nahlé

**Affiliations:** 1 Department of Cancer Biology and Genetics, Memorial Sloan-Kettering Cancer Center, New York, NY USA; 2 Department of Neurological Surgery, Vanderbilt University Medical Center, Nashville, TN USA; 3 Department of Cancer Biology, Vanderbilt University Medical Center, Nashville, TN USA; 4 Clinical Cooperation Unit Neuropathology, German Cancer Research Center (DKFZ), Heidelberg, Germany; 5 Division of Molecular Genetics, German Cancer Research Center (DKFZ), Heidelberg, Germany; 6 Present Address: Millipore Corporation, Billerica, MA USA

**Keywords:** Medulloblastoma, Cerebellum, Metabolism, PPARγ, Glycolysis, Sonic hedgehog, Cancer, Tumor metabolism

## Abstract

Sonic hedgehog (Shh) signaling is critical during development and its aberration is common across the spectrum of human malignancies. In the cerebellum, excessive activity of the Shh signaling pathway is associated with the devastating pediatric brain tumor medulloblastoma. We previously demonstrated that exaggerated de novo lipid synthesis is a hallmark of Shh-driven medulloblastoma and that hedgehog signaling inactivates the Rb/E2F tumor suppressor complex to promote lipogenesis. Indeed, such Shh-mediated metabolic reprogramming fuels tumor progression, in an E2F1- and FASN-dependent manner. Here, we show that the nutrient sensor PPARγ is a key component of the Shh metabolic network, particularly its regulation of glycolysis. Our data show that in primary cerebellar granule neural precursors (CGNPs), proposed medulloblastoma cells-of-origin, Shh stimulation elicits a marked induction of PPARγ alongside major glycolytic markers. This is also documented in the actively proliferating Shh-responsive CGNPs in the developing cerebellum, and PPARγ expression is strikingly elevated in Shh-driven medulloblastoma in vivo. Importantly, pharmacological blockade of PPARγ and/or Rb inactivation inhibits CGNP proliferation, drives medulloblastoma cell death and extends survival of medulloblastoma-bearing animals in vivo. This coupling of mitogenic Shh signaling to a major nutrient sensor and metabolic transcriptional regulator define a novel mechanism through which Shh signaling engages the nutrient sensing machinery in brain cancer, controls the cell cycle, and regulates the glycolytic index. This also reveals a dominant role of Shh in the etiology of glucose metabolism in medulloblastoma and underscores the function of the Shh → E2F1 → PPARγ axis in altering substrate utilization patterns in brain cancers in favor of tumor growth. These findings emphasize the value of PPARγ downstream of Shh as a global therapeutic target in hedgehog-dependent and/or Rb-inactivated tumors.

**Electronic supplementary material:**

The online version of this article (doi:10.1007/s00401-012-0968-6) contains supplementary material, which is available to authorized users.

## Introduction

Medulloblastomas (MBs) remain the leading cause of death from solid cancers in children [30]. These predominantly pediatric tumors of cerebellar origin present in infants, children, and to a lesser extent in the adult population. Concordantly, MBs are associated with aberrations in signaling cascades critical for brain development, organ patterning, and cell-fate determination like those controlled by the Wnt (WNT) or the hedgehog (HH) pathway. As high as 30 % of total human MBs bear evidence of aberrant hedgehog pathway activation. In this context, loss of function mutations in Patched (*Ptch*) or activating mutations in Smoothened (*Smo*), both trans-membrane proteins and essential components of Shh signaling, have been identified in human medulloblastomas [32, 34, 37, 48, 49]. Mutations in cytoplasmic components of the hedgehog pathway such as *Suppressor of Fused* (*SuFu*) have also been described [42]. Importantly, ectopic activation of Shh signaling [35, 36], constitutive activation of Smo itself [14, 15], or Ptch loss-of-heterozygosity [10] are all sufficient, independently, to drive medulloblastoma formation in vivo. Consistently, global inhibition of the Shh pathway at the level of its receptors, while transiently beneficial in MB patients, remains confounded by a rapid development of drug resistance, a trait also conserved in hedgehog-associated tumor-bearing mice [50]. Understanding the complexity of Shh signaling and the corresponding oncogenic network downstream of hedgehog is as such critical for developing effective and specific nodes for therapeutic manipulation.

In a recent study, we have demonstrated that hedgehog-driven MBs are distinguished by a dramatic accumulation of neutral lipids, underscoring these tumors’ metabolic, markedly lipogenic nature [2]. Such lipogenic features are similar to those described in gliomas with EGF receptor mutations [11] or a number of other human malignancies including cancer of the breast, lung, and prostate [25]. Importantly, we demonstrated mechanistically that the effect of Shh on lipogenic metabolism proceeds through a multi-step program involving the inactivation of the tumor suppressor Rb and the induction of its negative target E2F1, a key transcriptional regulator of proliferation networks [16, 27, 47]. Indeed, Shh-dependent induction of E2F1 promotes de novo lipid synthesis, induces the key enzyme Fatty Acid synthase (FASN), and primes the lipogenic machinery for a bioenergetics environment conducive to rapid proliferation. Impairing E2F activity and/or targeting de novo lipid synthesis with pharmacological means produced effective, reproducible therapeutic outcomes in MBs-bearing animals in vivo. These non-invasive approaches are especially promising for current MB treatments that damage the still-developing brain and result in catastrophic life-long side effects [31]. It will be insightful to decipher the key components regulating the metabolic machinery, in particular, those promoting the acquisition of permissive substrate utilization patterns that fuel disease progression and enable malignant transformation.

It is widely established that adaptations to perceived stress conditions are hallmarks of proper functioning in systemic physiology. In fact, reciprocity in coordinated pathways of glucose and lipid metabolism and flexible metabolic shifts are common with dietary flux conditions including starvation, fasting or postprandial states [39–41]. Such exquisite capacity for nutrient sensing is attributed, broadly speaking, to a complex web of signaling events. This includes (i) intracellular messengers like cAMP and associated kinases, (ii) mitochondrial enzymes responsive to acute changes in biochemical ratios such acetyl-CoA/CoA and NADH/NAD+ ratios, (iii) hormones that recruit specific substrate receptors like Glut4, and/or (iv) transcription factors that could alters metabolic patterns and substrate utilization at multiple, programmatic levels. A particular class of transcription factors with such capacity for reprogramming cellular responses and dictating nutrient sensing and signaling events are the fatty-acid activated peroxisome proliferator-activated receptors (PPARs). PPARs can elicit chronic, sustainable metabolic shifts through their transcriptional effects on metabolic enzyme gene expression. It is established that PPARγ activation confers significant benefit and ameliorate insulin sensitivity in vivo and ex vivo.

Here, we demonstrate the existence of a novel mechanism connecting Shh signaling to the PPARγ transcriptional machinery in neural precursors and in Shh-driven medulloblastoma. We show that Shh recruits the steroid nuclear receptor PPARγ, a key element in the nutrient sensing network, to reprogram cellular metabolism including the regulation of glycolysis proper. Indeed, Shh-dependent control of PPARγ dramatically activates the glycolytic modulators Glut4, hexokinase II (HKII) and pyruvate kinase M2 (PKM2), and promote glucose uptake in tumors in vivo. Importantly, the mechanism linking Shh to PPARγ and its aforementioned targets requires E2F1 in vivo and ex vivo*.* In addition, this process is fully reversible with antagonizing PPARγ, an event that remarkably blunts tumor proliferation and extends survival of animals with medulloblastoma in vivo. In short, findings of this study provide critical insights into how vital and interlocked metabolic networks are altered or co-opted in Shh-driven MBs. The data also underscore the significant role of PPARγ downstream of Shh in dictating modes of substrate utilization and defining metabolic patterns in these tumors, and possibly other hedgehog-associated cancers. Given the fundamental role for PPARγ in nutrient sensing and the integration of dietary signals on the one hand and Shh functions in a myriad of biological activities like proliferation and self-renewal on the other hand, such link highlights the interplay between cell cycle regulators and the fuel processing machinery. Furthermore, these results are equally relevant to the pathophysiology of chronic diseases where glucose metabolism is dysregulated, particularly where sonic hedgehog activities are also implicated [21, 22]. Finally, considering the central role of the Rb-E2F complex in survival and proliferation and the widespread Rb pathway mutations found in human cancers, our findings could assist in better defining the etiology of tumor metabolism, in particular the perplexing phenotype of aerobic glycolysis and the lack of efficient mitochondrial oxidation of glucose despite the ostensible need for ATP in hyperproliferative cells. The requirement for E2F1 in Shh-regulated PPARγ dependent-glycolysis, as demonstrated, highlights its engagement in metabolic core fluxes and underscores the critical role for the Rb/E2F complex in metabolic reprogramming of medulloblastomas and their cells-of-origin.

## Materials and methods

### Animal studies

Wild type C57-BL6 mice and NeuroD2-SmoA1 mice bearing tumors were administered 10 % dimethyl sulfoxide (DMSO, control), olomoucine (Calbiochem) at 6 mg/kg daily or GW9662 (Cayman Chemicals) at 10 mg/kg daily by intraperitoneal (i.p.) injection. Wild-type C57/BL6 mice, NeuroD2-SmoA1 mice, and E2F1-null mice were obtained from Jackson Laboratory (Bar Harbor, ME, USA) and typically were 4–5 months of age when demonstrating the signs of a tumor (ataxia, etc.).

### Cell culture

Cerebellar granule neural precursor cultures were generated as previously described in detail [19]. The cell culture media consisted of DMEM-F12 (Gibco, Grand Island, NY) and 1× dilutions of N2 supplement (as 100× from Gibco), Penicillin Streptomycin (100× from Gibco) and potassium chloride (2.5 M is a 100× stock). P4 and P5 wild type C57/BL6 pups were killed and the cerebellum extracted and placed into 1× Hanks buffered saline solution (HBSS) (Gibco) with 6 gm/L of glucose at a pH of 7.4. The HBSS was aspirated under a tissue hood and trypsin (Gibco) was added with 1 mg/ml of DNAse. The trypsin was then inactivated with the media containing 10 % FCS and the cerebella titurated twice. Following a 5 min spin at 1,500 rpm, the pellet was resuspended in 10 % FCS media. Cells were then plated on polyornithine-coated six well plates at a concentration of 3 million cells/mL in 1 mL of the 10 % FCS media for 6–12 h. The media were then replaced with FCS free media and given their treatments and incubated for 24 h before protein lysis. Where indicated Shh (R&D Systems, Minneapolis, MN, USA) was used at a concentration of 3 μg/ml, cyclopamine (R&D Systems) was used at 1 μg/ml and GW9662 (Cayman Chemical, Ann Arbor, MI, USA) was used at 50 or 100 nM.

### Western blotting

Protein extracts were prepared as previously described [20]. A total of 40 μg of murine cerebellum, medulloblastoma, or CGNP protein extract were run on 8–12 % SDS–polyacrylamide gels and transferred to a PVDF membrane (Millipore, Billerica, MA, USA). The blots were blocked and incubated with primary antibodies in 5 % milk in Tris-buffered saline-Tween (TBS-T) overnight in 4 °C. Blots were washed three times and incubated with secondary antibodies in 5 % milk in TBS-T for 2 h in room temperature. After washing, the signals were developed using the enhanced chemiluminescence method (GE Healthcare, Piscataway, NJ, USA) and the membranes were exposed to Kodak Biomax film. Primary antibodies were: E2F1 (Cell Signaling, Danvers, MA, USA), PPARγ (E-8; Santa Cruz Biotechnology, Santa Cruz, CA, USA), FASN (Cell Signaling), Hexokinase I (Cell Signaling), Hexokinase II (Cell Signaling), Glut4 (Cell Signaling), PKM2 (Cell Signaling), Cyclin D2 (M-20; Santa Cruz) and β-tubulin (Sigma). Horseradish peroxidase-conjugated secondary antibodies were: goat anti-rabbit IgG (H–L; Thermo Scientific, Rockford, IL, USA) and donkey anti-mouse IgG (H–L; Jackson Immuno-Research, West Grove, PA, USA). Primary antibodies were diluted to 1:1000 for incubation in 5 % milk in TBS-T with the exception of β-tubulin, which was 1:5,000 while the secondary antibodies were at 1:10,000 as recommended by their manufacturers.

### Lentivirus production and infection

E2F1 shRNA and scrambled control lentivirus constructs were obtained from The RNAi Consortium (Sigma, St Louis MO). Each construct was transfected into the Pzp53med cell line, which was derived from a medulloblastoma arising in a Ptc^+/−^/p53^−/−^ mouse [1], and western blotting was used to determine which shRNAs effectively and specifically targeted E2F1. These constructs were used to prepare lentiviruses. 293T cells grown in 10 % FBS Dulbecco’s modified Eagle’s medium (Gibco) were transfected with E2F1 or scrambled control shRNA lentiviral vectors and MISSION lentiviral packaging mix (Sigma) using Fugene 6 in serum-free OPTI-MEM medium (Invitrogen, Carlsbad, CA, USA). Lentiviral supernatants were collected 48-h post-transfection and filtered through a 0.45 μ filter, then pooled. For infection, Shh-treated CGNPs were exposed to the lentiviral supernatant for 4 h. Control scrambled shRNA lentivirus constructs were used to determine specificity for E2F1 and to rule out off-target effects or non-specific results because of the process of virus infection. The lentiviruses were aspirated and replaced with serum-free CGNP medium (above) containing Shh. The cells were lysed 48-h post-infection.

### Immunostaining

Tissues were fixed in 4 % para-formaldehyde and paraffin-embedded as previously described [3]. After dehydration steps and sectioning, tissue slides were washed with 1× phosphate buffered saline (PBS) and permeabilized in 1 % TritonX-100 for 5 min. Slides were then blocked in 5 % goat serum in PBS-Tween (1× PBS and 0.1 % TritonX-100) for 1 h in room temperature, washed once with 1× PBS, and incubated with primary antibody in 2.5 % goat serum (in PBS-Tween) overnight at 4 °C. They were washed three times with 1× PBS and incubated with secondary antibody for 2 h at room temperature, then washed and mounted in 40-6-diamidino-2-phenylindole-containing mounting medium (Vector Labs, Burlingame, CA, USA). Primary antibodies used were: E2F1 (H-137; Santa Cruz), PPARγ (E-8; Santa Cruz), FASN (Cell Signaling, Danvers MA), Hexokinase I (Cell Signaling), Hexokinase II (Cell Signaling), Glut4 (Cell Signaling), PKM2 (Cell Signaling), P-Histone H3 (Cell Signaling), and Ki67 (Vector Labs, Burlingame CA). Secondary fluorescent-tagged antibodies were: Alexa Fluor goat anti-rabbit 488/594 (Invitrogen, Carlsbad, CA) and Alexa Fluor goat anti-mouse 488/594 (Invitrogen).

### Image capturing

Immunostaining performed on tissue sections was visualized using a Leica DM5000B microscope and images were captured with Leica FW400 software (Leica Microsystems, Bannockburn, IL, USA).

### Oil Red O staining

Oil Red O staining of neutral lipids in NeuroD2-SmoA1 medulloblastoma frozen sections was performed by the histology core in the Department of Pathology and Laboratory Medicine in the Hospital for Special Surgery and by the microcytometry core facility at Memorial Sloan–Kettering Cancer Center according to established protocols.

### PET scanning

For fluorine-18 fluoro-deoxyglucose (FDG) studies using the Focus 120 microPET small-animal PET scanner (Concorde Microsystems, Knoxville, TN), animals were fasted for 6 h prior to injection of the radiotracer; access to water was permitted. Mice were bolus injected via lateral tail vein with 300 mCi of the PET radiotracer FDG in ~200 ml of normal saline. At ~45-min post-injection, the mouse was first anesthetized by inhalation of a 2 % isofluorane/oxygen gas mixture (flow rate of 2 L/min.) and placed on the scanner bed in the prone position. The acquisition is set up for ~5 min using a photopeak energy window of 350–750 keV, and a coincidence timing window of 6 nsec—and launched using the microPET’s microPET Manager™ computer software. Data are acquired in list mode. The resulting data are sorted into 2D histograms by Fourier re-binning and transverse images are reconstructed by filtered back-projection (FBP) into a 3D imaging matrix using the system Nyquist frequency as the cut-off. The image data are normalized to correct for non-uniformity of response of the microPET, dead-time count losses, positron branching ratio, and physical decay to the time of injection, attenuation, scatter, or partial-volume averaging corrections are generally not applied. An empirically determined system calibration factor (in units of mCi/mL/cps/voxel) for mice is used to convert voxel count rates to activity concentrations, and the resulting image data are then normalized to the administered activity to parameterize images in terms of percent of the injected doses per gram (%ID/gm). Manually drawn 2D regions-of-interest (ROIs) or 3D volumes-of-interest (VOIs) are used to determined the mean and maximum %ID/g (decay corrected to the time of injection) in tumors and various organs using ASIPro VM™ computer software (Concorde Microsystems).

### Cell viability

Cell viability was determined with the cell Titer Glo assay (Promega, Madison, WI) which utilizes luminescent reagents reacting with ATP yielding a quantitative expression of living cells in the sample expressed as a relative luminescence between samples. This luminescence was measured with the Glo Max Multi + (Promega) plate reader. Statistical analysis was accomplished with SAS software package (SAS Inst. Inc., N.C., USA). Significant differences between mean were determined by ANOVA procedure test and a *P* value of <0.05 was considered to be statistically significant. The results are expressed as the mean ± SD from quadruplicate test data.

## Results

### Marked deregulation of PPARγ in Shh-driven mouse medulloblastomas

**Fig. 1:**
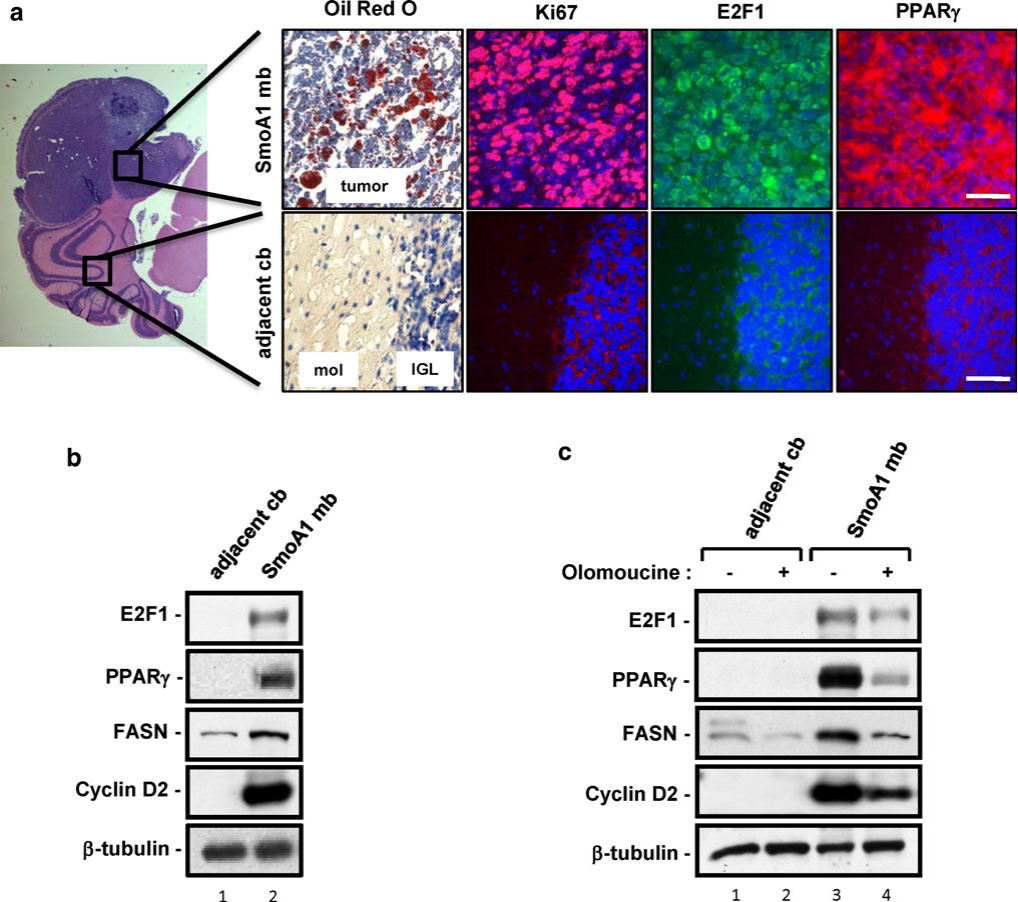
Shh-induced mouse medulloblastomas have robust lipogenesis, increased levels of E2F1 and PPARγ and cell proliferation. **a** Triglyceride accumulation was analyzed in a *NeuroD2*-SmoA1 medulloblastoma using Oil Red O staining; neutral lipids appear as red droplets. *NeuroD2*-SmoA1 medulloblastoma (*top row*) and adjacent non-tumorous cerebellum (*bottom row*) were subjected to immunofluorescence analyses for E2F1, PPARγ, and the proliferation marker Ki67. Primary and secondary antibodies were used at concentrations of 1:100. *Leftmost panel* shows hematoxylin and eosin staining (H&E) staining of the tumor and cerebellar molecular layer (*mol*) and inner granule layer (*IGL*). The non-tumorous cerebellum immunofluorescence focuses on the boundary between the IGL and molecular layer demonstrated in the H&E staining. Magnification: ×1.25 (*leftmost panel*), ×40 (*other images*). *Bars* 16 μM. *Panels* demonstrate the typical outcome from 5 different tumor and adjoining cerebellum specimens all paraffin embedded, sectioned, and subjected to immunofluorescence for the aforementioned antibodies. Additionally, the Oil red oil represents a typical outcome based on the staining of frozen sections from 5 different specimens. **b** Protein lysates were prepared from *NeuroD2*-SmoA1 medulloblastoma and normal cerebellum adjacent to the tumor, and then analyzed by western blotting for PPARγ, lipogenic marker (FASN) and proliferation markers (E2F1 and cyclin D2). 40 μg protein/lane was loaded. Data presented are typical for all 5 sets of tumor and cerebellum samples that were studied. **c** Western blot analysis of proteins regulating proliferation and lipogenesis in adjacent non-tumor cerebellum or medulloblastomas from *NeuroD2*-SmoA1 mice treated with DMSO (−) or cdk inhibitor olomoucine (+). The results shown represent a typical outcome amongst the control and experimental groups of mice

We have previously reported that the subclass of medulloblastomas resulting from aberrant activation of Shh signaling [14, 15] is marked by strikingly high levels of de novo fatty acid synthesis, as determined by staining for neutral lipids (Fig. 1a, top left, Oil Red O staining), compared to the adjacent non-tumorigenic cerebellar tissue (bottom left). This exaggerated lipogenesis is associated with high levels of proliferation as indicated by Ki67 immunofluorescence, and increased cdk activity, leading to Rb inactivation and induction of E2F1 (Fig. 1a, b). Indeed, we have also shown previously in that context that E2F1 is required for expression of the key lipogenic enzymes including fatty acid synthase (FASN) and acetyl CoA carboxylase (ACC), and that abrogation of E2F1 activity in vivo results in impaired lipogenesis in tumors. This firmly linked E2F1 activity downstream of Shh to de novo lipid synthesis in medulloblastoma and their proposed cells-of-origins.

PPARγ is a pivotal transcription factor that is triggered by fatty acid ligands and a key regulator of lipogenesis. Here, using immunostaining and blotting techniques in mouse NeuroD2-SmoA1 medulloblastomas, we detected very robust levels of PPARγ in the tumors as opposed to the adjacent non-tumor cerebellar tissues (Fig. 1a, b). We next examined whether in medulloblastomas expression of PPARγ, like that of FASN, requires E2F1 activity. PPARγ has been reported to be transcriptionally controlled by E2F1 in adipocytes [7]. We treated medulloblastoma-bearing mice with the cdk inhibitor olomoucine (6 mg/kg) for 10 days. As we have previously reported, this treatment regimen markedly prolongs survival of tumor-bearing mice and is associated with reduced E2F1 and FASN expression (Fig. 1c) and with diminished proliferation in the tumors, as indicated by reduced levels of cyclin D2 (Fig. 1c). Indeed, olomoucine treatment resulted in a striking reduction of PPARγ levels in the NeuroD2-SmoA1 medulloblastomas (Fig. 1c). These results suggest that PPARγ lies downstream of the Shh → E2F1 axis in Shh-driven medulloblastomas and further, that investigating pathways downstream of PPARγ could lead to a better insight into the biology of medulloblastoma and their cancer associated metabolic patterns.

### Glycolytic enzymes are elevated in NeuroD2-SmoA1 medulloblastomas

**Fig. 2:**
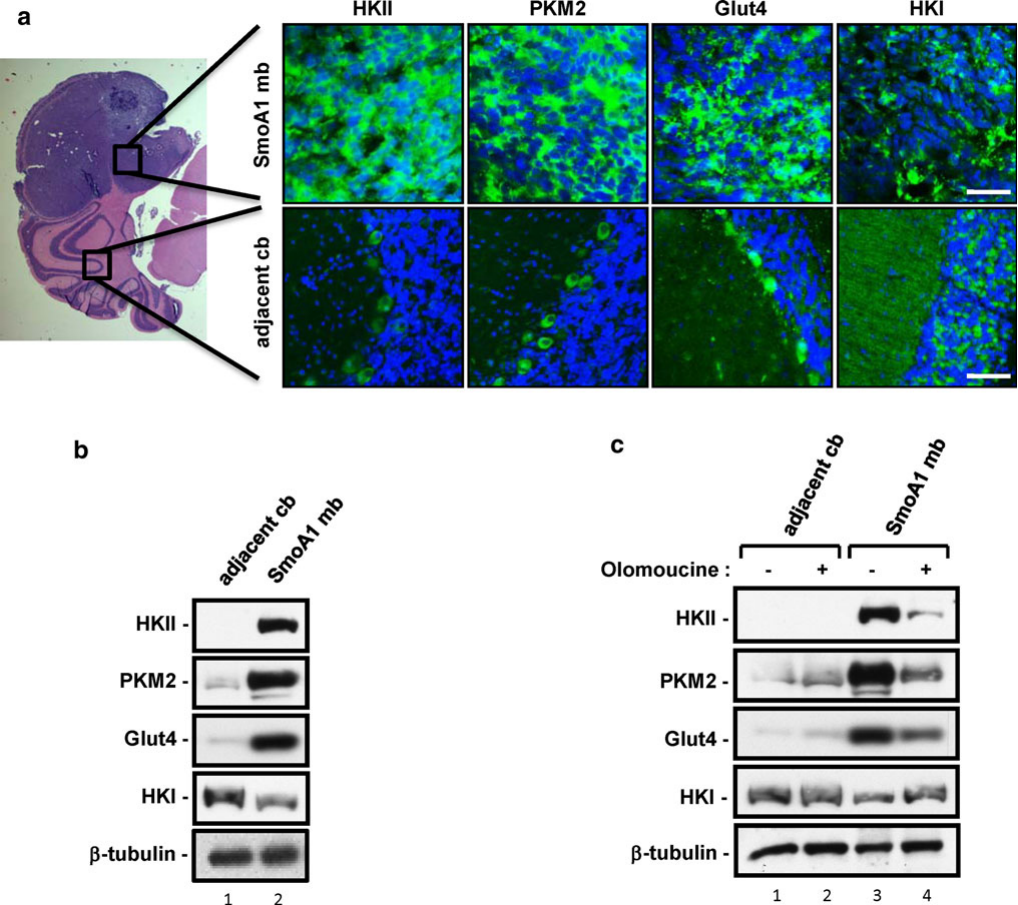
Shh-induced mouse medulloblastomas have high levels of glycolytic markers of hexokinase II, pyruvate kinase M2, and glucose transporter-4. **a** *NeuroD2*-SmoA1 medulloblastoma (*top row*) and adjacent non-tumor cerebellum (*bottom row*) were subjected to immunofluorescence analyses for glycolysis (HKI, HKII, Glut4, and PKM2). *Leftmost panel* shows hematoxylin and eosin staining (H&E) staining of the tumor and cerebellar molecular layer (*mol*) and inner granule layer (*IGL*). Magnification: ×1.25 (*leftmost panel*), ×40 (*other images*). *Bars* 16 μM. Results shown are typical for the 5 different tumor and adjoining cerebellum samples used for immunofluorescence in this study. **b** Protein lysates were prepared from *NeuroD2*-SmoA1 medulloblastoma and normal cerebellum adjacent to the tumor, and then analyzed by western blotting for glycolysis. 40 μg protein/lane was loaded. The data shown were repeated with multiple sets of tumor and cerebellum from different *NeuroD2*-*SmoA1* mice exhibiting tumors. **c** Western blot analysis of proteins regulating glycolysis in adjacent non-tumor cerebellum or medulloblastomas from *NeuroD2*-SmoA1 mice treated with DMSO (−) or cdk inhibitor olomoucine (+). The same protein lysates from Fig. 1c were blotted for glycolytic markers and this is a typical outcome of those blots

Substrate utilization pathways are functionally intertwined. Cells in anabolic tissues have a large capacity to generate anew and store fats from excessive glucose influx. We have shown that Shh-driven medulloblastomas and their proposed cells-of-origin, cerebellar granule neuron precursors (CGNPs), exhibit high levels of lipogenesis, and here we report that these tumors feature increased PPARγ. In order to determine whether glycolysis is increased in NeuroD2-SmoA1 medulloblastomas, we carried out immunostaining and western blot analysis of medulloblastomas and adjacent, non-tumorous cerebellum. As shown in Fig. 2a, b, the medulloblastomas are marked by a robust accumulation of many key glycolytic enzymes, including hexokinase II (HKII), pyruvate kinase M2 (PKM2), and glucose transporter type 4 (Glut4), which control glucose transport, especially into muscle and adipocytes in response to insulin stimulation. Notably such patterns appear to be specific to HKII as hexokinase I (HKI) levels were higher in normal cerebellar tissue than in the medulloblastomas.

We next asked whether the expression of these glycolytic enzymes, like that of lipogenic markers, is dependent upon Rb inactivation and E2F1 activity. To this end we used western blot analysis of cerebellar tissue and medulloblastomas prepared from DMSO- or olomoucine-treated mice. As shown in Fig. 2c, levels of HKII, PKM2, and Glut4 were all reduced in olomoucine-treated tumors, while levels of HKI were up-regulated in the olomoucine-treated medulloblastomas. These results indicate that the glycolytic markers are associated with E2F1 activity and proliferation, and further suggest that HKI plays a role distinct from glycolysis in differentiated (e.g. normal cerebellum) or non-proliferating tissue (e.g. olomoucine-treated medulloblastoma).

### E2F1 is required for PPARγ and the glycolytic markers expression in neural precursors of the developing cerebellum

**Fig. 3:**
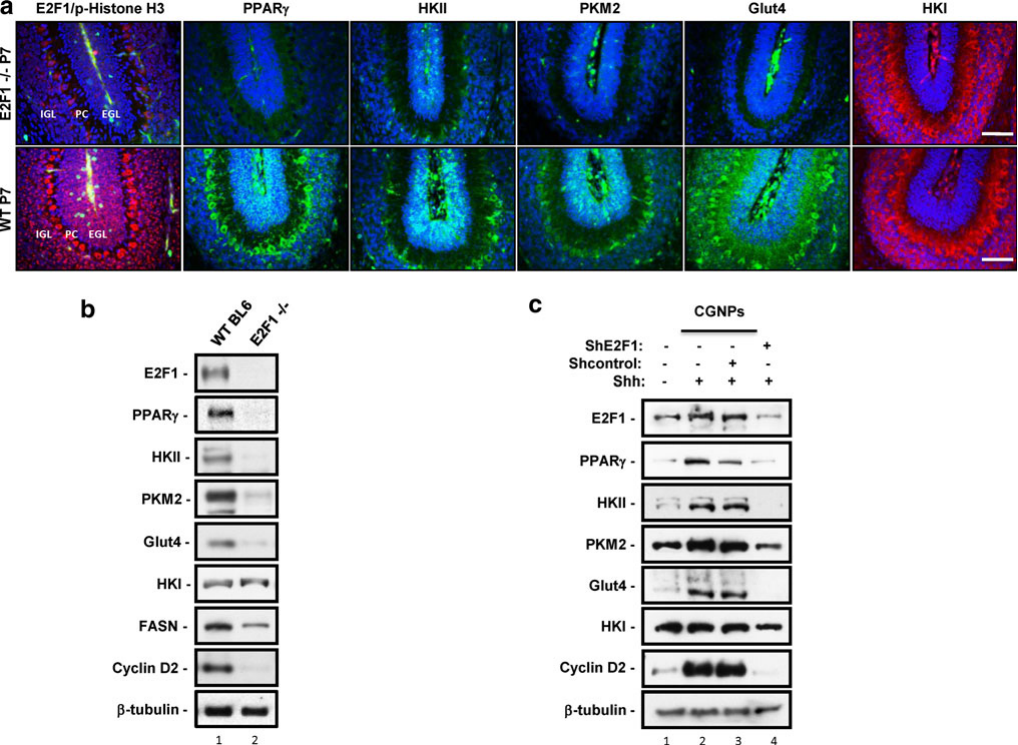
Loss of E2F1 in mice ablates PPARγ, and key markers of de novo lipogenesis, glycolysis, and cell proliferation. **a** PN 7 sagittal sections of wild-type (*bottom row*) and E2F1-null (*top row*) cerebella were immunostained for the glycolysis-related proteins Glut4, muscle pyruvate kinase 2 (PKM2), hexokinase 1 and 2 (HKI; HKII) as well as PPARγ, and E2F1 with the proliferation marker phospho-histone H3. *First column panel* shows cerebellar inner granule layer (*IGL*), Purkinje cells (*PC*), and external granule layer (*EGL*). Magnification: ×40. *Bars* 32 μM. These slides are indicative of several specimens from the wildtype and E2F null groups that were analyzed with IF. **b** Cerebella were collected from wild-type or E2F1-null PN 5 pups, then analyzed by western blotting for levels of E2F1, PPARγ, glycolysis proteins (HKI, HKII, PKM2, and Glut4), the lipogenic marker fatty acid synthase (FASN), and proliferation marker cyclin D2. Results are typical of several sets of protein lysates that were collected. **c** Western blot analysis of E2F1, PPARγ, glycolysis (HKI, HKII, PKM2, Glut4) and proliferation marker cyclin D2 in CGNPs treated with DMSO vehicle, Shh or Shh in the presence of lentiviruses carrying shRNAs targeting E2F1 or a scrambled control shRNA virus

Medulloblastomas associated with increased activity of the Shh pathway have been proposed to arise from proliferating neural precursors in the developing cerebellum [6]. During perinatal development, these cells proliferate rapidly in the external germinal layer (EGL) response to Shh secreted from the underlying Purkinje cells (PN) [4, 44, 45]. Upon exiting the cell cycle they migrate inward to the internal granule layer (IGL), where they undergo terminal differentiation. Shh-associated medulloblastomas share many characteristics of these cerebellar granule neuron precursors (CGNPs), including high expression of N-myc, microRNA miR 17/92, increased lipogenesis, and as we previously reported and show again here (Fig. 3a, b) elevated E2F1 [2, 29, 33]. To determine whether increased glycolysis is also a shared feature of Shh-associated medulloblastomas and CGNPs, and whether this is, like lipogenesis, an E2F1-dependent event, we carried out immunofluorescence and western blot analysis of wild-type and E2F1-null mouse cerebella at postnatal day 7 (PN7), when CGNP proliferation is at its peak.

As shown in Fig. 3a (bottom panels) and in Fig. 3b, PPARγ is found in the EGL, as are HKII, PKM2, and Glut4. We also observed some expression of Glut4 in the IGL. In contrast, HKI was absent from the EGL and was highly expressed in the IGL, where differentiating CGNPs are found. Ablation of E2F1 resulted in loss of expression of PPARγ, HKII, PKM2, and Glut4, but did not affect levels of HKI. These results suggest that in the developing cerebellum, as in medulloblastoma, PPARγ and glycolytic markers lie downstream of Sonic hedgehog and E2F1.

We next asked whether CGNPs carry out glycolysis in vitro, and whether acute manipulation of E2F1 abrogates this, as it does lipogenesis [2]. We established primary cultures of CGNPs from PN 4/5 neonatal mice. These cells are grown in the absence of serum; addition of exogenous Shh to their medium promotes ongoing proliferation, while in its absence the cells leave the cell cycle and differentiate as they would in vivo. We infected Shh-treated CGNPs with lentiviruses carrying a control, scrambled short hairpin RNA (shRNA) sequence or a shRNA sequence targeting E2F1 for knock-down. As shown in Fig. 3c and as previously reported, these lentiviruses effectively knocked down E2F1, while infection with the control shRNA had no effect on E2F1 levels. In the presence of Shh, CGNPs have increased levels of PPARγ, HKII, PKM2, and Glut4, consistent with our observations in vivo. Levels of HKI were unaffected by the presence of Shh. Infection with the scrambled shRNA results in a slight reduction of PPARγ levels but has no effect on levels of the glycolytic markers. In contrast, E2F1 knock-down effectively abrogated expression of PPARγ and the associated glycolytic markers. Taken together, the results of our in vivo analysis and in vitro manipulation of E2F1 indicate that in Shh-associated mouse medulloblastomas and in proliferating CGNPs, glycolysis is elevated in an E2F1-dependent manner and its regulation functionally lie downstream of PPARγ.

### PPARγ activity is required for the expression of glycolytic markers in CGNPs

**Fig. 4:**
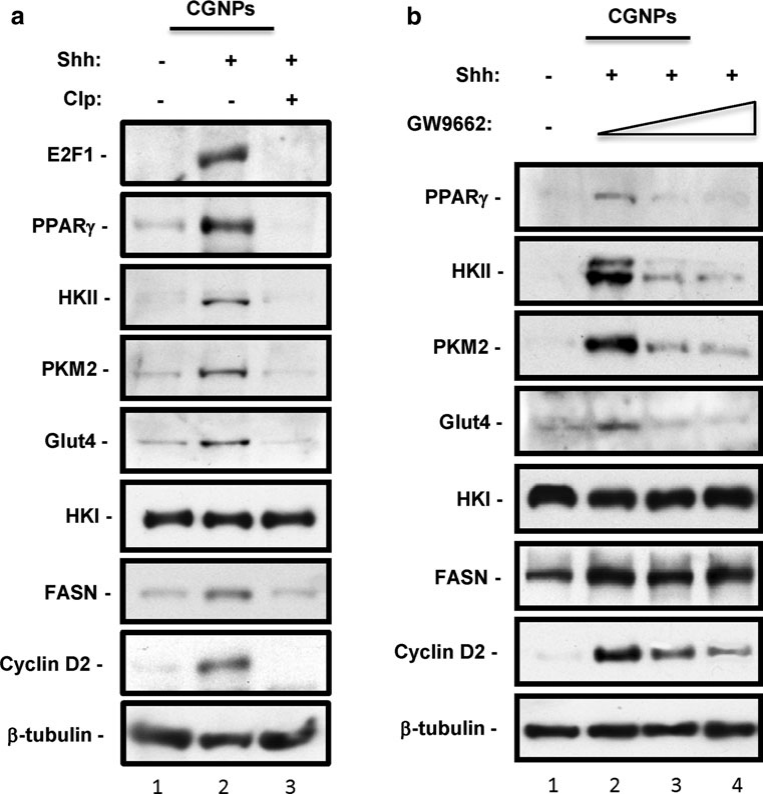
Glycolysis in CGNPs is Shh- and PPARγ-dependent. **a** CGNP cultures were prepared for western blot analysis from PN 4/5 mice and incubated with vehicle (Veh), Shh (3 μg/ml) and/or Smoothened inhibitor cyclopamine (10 μg/ml). The lysates were blotted for the glycolytic and proliferative proteins. The presented work was repeated with several sets of CGNP lysates from PN 4/5 mice. **b** CGNP cultures were prepared for western blot analysis from PN 4/5 mice and incubated with vehicle (Veh), Shh (3 μg/ml) and/or PPARγ antagonist GW9662 in increasing doses of 0, 50, and 100 nM

CGNPs require Shh pathway activity for proliferation as well as for E2F1 expression. To establish that PPARγ and glycolysis are regulated by Shh upstream of E2F1, we cultured CGNPs in the presence of Shh or in the presence of Shh and cyclopamine, which inhibits Smoothened and blocks Shh signaling. As we have shown previously, cyclopamine treatment blunts the accumulation of E2F1 and FASN, and reduced proliferation as determined by cyclin D2 levels (Fig. 4a). Exposure to cyclopamine had no effect on HKI. However, in the presence of cyclopamine levels of PPARγ, HKII, PKM2, and Glut4 were strongly reduced, consistent with Shh signaling being required for E2F1 activation and induction of glycolysis, and indicating that glycolysis is a property associated with CGNP proliferation.

Thus far, our results indicate a requirement for E2F1 in the regulation of PPARγ and the enzymes promoting glycolysis. As shown in Fig. 3, PPARγ and glycolytic enzymes lie downstream of E2F1. We wished to determine whether the activity of PPARγ is required for glycolytic marker up-regulation in CGNPs. It is known that treatment of cells with PPARγ agonists such as rosiglitazone is associated with induction of glycolytic enzymes including Glut4 [8], although the precise mechanism through which this takes place is unknown. We carried out dose–response studies with the PPARγ-specific inhibitor GW9662, which has been used in vitro and in vivo to inhibit PPARγ and displays no activity towards other PPAR family members [23, 28]. As shown in Fig. 4b, treatment of CGNPs with 50 or 100 nM GW-9662 resulted in loss of detectable PPARγ, which was remarkably associated with reduced HKII, PKM2, and Glut4. Importantly, GW9662 treatment had no effect on HKI and also did not reduce levels of FASN, indicating that although both FASN and PPARγ lie downstream of E2F1, PPARγ and FASN are regulated independently. Notably, GW9662 treatment also reduced CGNP proliferation as indicated by diminished cyclin D2 levels. Thus, PPARγ activity is required for glycolysis and ongoing cell division in CGNPs.

### Inhibition of PPARγ impairs glycolysis in medulloblastoma and prolongs survival of tumor-bearing mice

**Fig. 5:**
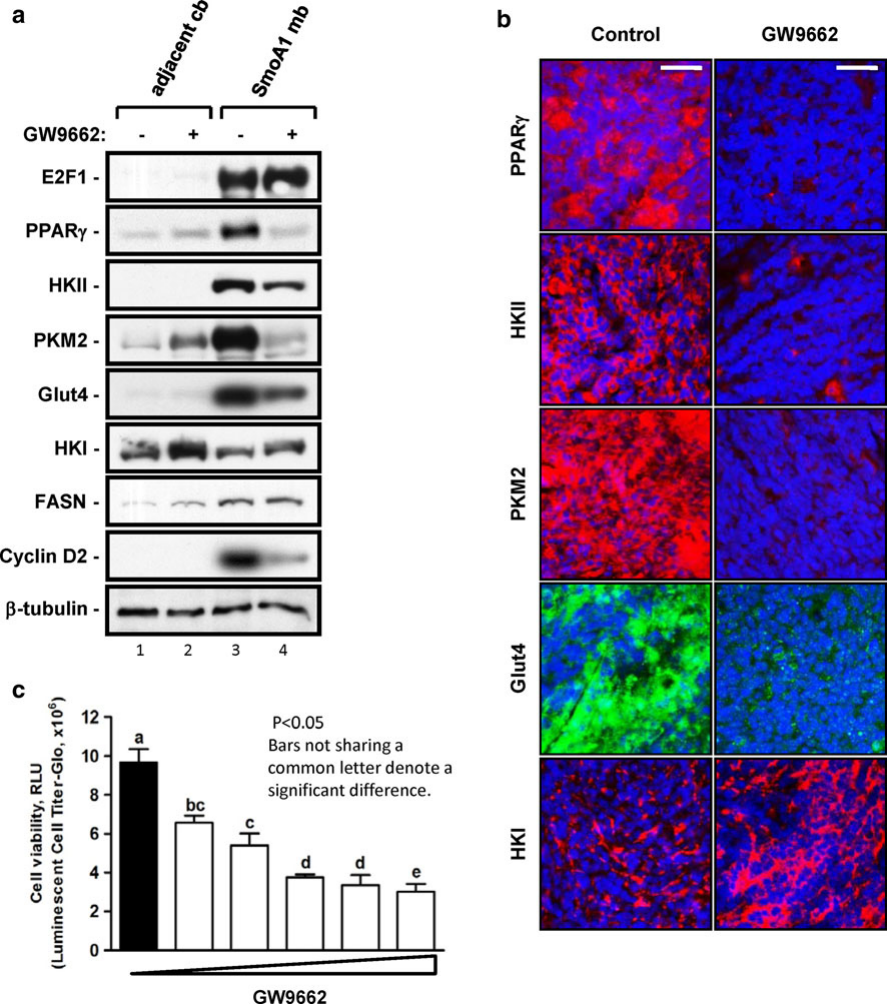
Treatment of medulloblastoma-bearing mice with PPARγ antagonist GW9662 impairs glycolysis in tumor in vivo. **a** Western blot analysis for E2F1, PPARγ, glycolysis (HKI, HKII, PKM2, and Glut4), lipid synthesis (FASN) and proliferation (cyclin D2) in samples of adjacent cerebella and medulloblastomas from vehicle (DMSO)-treated (−) or GW9662-treated (+) mice. The results shown are typical for several control and experimental sets of mice. **b** Immunostaining in adjacent cerebellum and medulloblastomas in vehicle- or GW9662-treated mice. Slides from the tissue of several untreated and treated *NeuroD2*-*SmoA* mice exhibiting medulloblastomas were analyzed and the presented results were typical. Magnification, ×40. *Bars* 16 μM. **c** Effects of increasing doses of the PPARγ antagonist GW9662 (0, 0.01, 0.02, 0.05, 0.07, 0.1 μM) for 24 h on pzp53med cell viability as determined by cell Titer-Glo assay, a bioluminescent analysis based on the presence of ATP. *Each bar* in the bioluminescence graph represents the average of separate quadruplicate determinations with *error bars* showing the standard deviation of the mean. *Bars* not sharing common letter are significantly different at *P* < 0.05

Our data in CGNPs show that PPARγ is associated with a proliferative state, and that PPARγ positively regulates glycolytic markers. Many tumor cells are known to exhibit high levels of glycolysis, and NeuroD2-SmoA1 medulloblastomas are consistently highly proliferative. We wished to determine whether PPARγ inhibition could impair glycolysis and affect proliferation in these tumors. We treated medulloblastoma-bearing NeuroD2-SmoA1 mice with GW9662 or its vehicle DMSO for up to 10 days, then harvested the tumors for western blot and immunofluorescence analysis. As shown in Fig. 5a, GW9662 treatment had no impact on the level of E2F1 or FASN, but sharply reduced PPARγ protein levels (Fig. 5a, b), consistent with PPARγ lying downstream of E2F1 and in a parallel pathway to FASN. GW9662 treatment also reduced levels of glycolytic markers and cyclin D2 protein, but had no effect on HKI, in keeping with our in vitro results in CGNPs. When tested in the pzp53 MB cell line, GW9662 treatment was very effective in killing cancer cells in a dose-dependent manner (Fig. 5c), as show by a luminescence viability assay (Cell Titer-Glo).

**Fig. 6:**
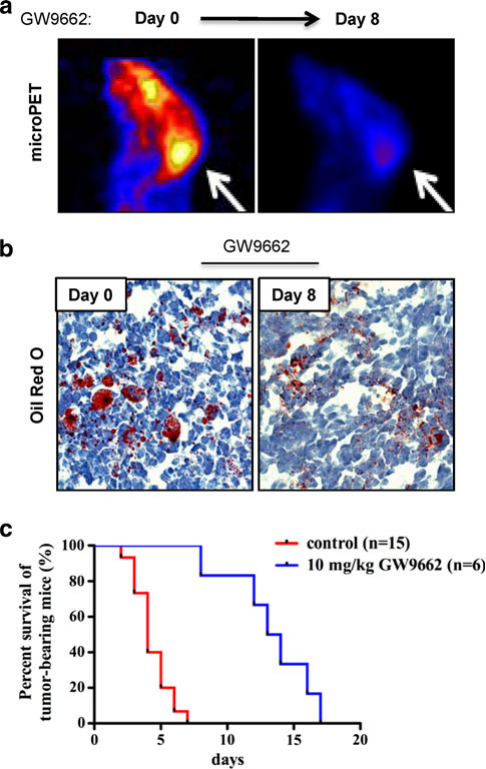
Treatment of medulloblastoma-bearing mice with PPARγ antagonist GW9662 prolongs survival and reduces glucose uptake in vivo. **a** FDG-PET scan of medulloblastoma-bearing *NeuroD2*-*SmoA1* mouse before and after treatment with the PPARγ antagonist GW9662 (10 mg/kg, intraperitoneal injection daily over the 8-study day period). **b** A representative Oil red O staining of medulloblastoma sections from an untreated mouse (*day 0*) and a counterpart treated with GW9662 for 8 days. **c** Kaplan–Meyer survival curve of *NeuroD2*-SmoA1 medulloblastoma-bearing mice in days commencing with initiation of treatment with DMSO or GW9662.; *P value <0.0001, Mantel–Cox test analysis). Magnification, ×40. *Bars* 16 μM

Using FDG-PET analysis, we observed a striking reduction in glucose uptake in GW9662-treated mice (Fig. 6a). This correlates with a functional effect of PPARγ inhibition on de novo lipid synthesis (Fig. 6b). Finally, we assayed the effects of GW9662 treatment on survival of NeuroD2-SmoA1 medulloblastoma-bearing mice. As shown in Fig. 6c, from the time of tumor onset as determined by appearance of detectable symptoms such as head tilt and altered gait and confirmed by magnetic resonance imaging, the average length of survival of NeuroD2-SmoA1 animals treated with DMSO (*N* = 15) was approximately a week. In contrast, GW9662-treated mice (*N* = 6) lived for up to 16 days after tumor onset, a significant increase in survival. These striking results suggest a therapeutic potential for PPARγ inhibition in treating Shh-driven medulloblastomas.

## Discussion

Cross talk between independent yet intertwined signaling pathways of metabolism and cancer is currently the topic of increasing scrutiny. We reported earlier a direct transcriptional link between the Rb/E2F tumor suppressor complex and PDK4 [17], a critical nutrient sensor and a regulator of mitochondrial glucose oxidation. In more recent work, we demonstrated that excessive mitogenic signaling by Shh, a driver of medulloblastoma, engages the Rb/E2F tumor suppressor complex, activates the key enzyme fatty acid synthase (FASN) and drives lipogenesis through a novel, E2F1-regulated process [2]. FASN has also been linked to survival in glioma cells with activating EGFR mutations [11, 12] and other tumors [25]. Many transformed cells also exhibit reliance on glutaminolysis [5, 43], a process associated with the activity of the myc oncogene [9, 46]. In addition, expression of key tumor suppressors such as p53, PTEN and LKB1 has been reported to influence important regulators of cellular metabolism [18]. Indeed, many signaling pathways implicated in routine surveillance themselves go awry across the spectrum of human diseases. Loss of metabolic inflexibility for instance is a pathological feature in disorders like diabetic cardiomyopathy as well as in certain cancers, where tumor cells’ addiction to de novo lipid synthesis for instance appears to be a defining feature for proliferation and survival [2, 24].

In this study, we show that in medulloblastoma and CGNPs, the Shh pathway is coupled—through E2F1—to the steroid receptor PPARγ and the control of glycolysis, a vital cellular process. This coupling underscores the broad influence of the hedgehog-regulated metabolic network. It is significant given the role of PPARγ as a modulator of cellular homeostasis as well as a therapeutic target in many chronic diseases [26]. Indeed, PPARγ agonists are routinely used for the treatment of patients with diabetes or hyperglycemia and it is established that activation of PPARγ ameliorates insulin sensitivity in vivo and ex vivo. Nonetheless, the findings of this study reveal that antagonizing, not stimulating, PPARγ would have a practical therapeutic use in MBs. Indeed, inhibiting PPARγ was markedly beneficial in counteracting hyperproliferation, reducing tumor burden, and extending the survival of Shh-driven MBs in mice (Fig. 6). Of note, when examined for their PPARγ expression using tissue microarrays, marked PPARγ staining was detected in >50 % of the Shh subgroup (Supplementary data). Intriguingly high PPARγ staining was also present in other groups and prevalent in Group D samples. While more elaborate analysis (including demographic, gender, survival) in larger cohorts is needed to understand the role and function of PPARγ in human tumors, deregulation of this pathway appears to be widespread in human MB.

The aforementioned role for the E2F1 → PPARγ axis as a potent effector of hedgehog signaling in modulating the glycolytic index has also numerous implications. In addition to presenting a viable therapeutic avenue in this pediatric brain tumor, it helps explain tumor etiologies associated with hedgehog and/or Rb/E2F deregulation. Elevated glucose uptake and hexokinase II activity have been characteristic features correlated with poor prognosis in a wide spectrum of malignancies. These features are routinely exploited in diagnostic imaging techniques like fluorodeoxyglucose (FDG)-positron emission tomography [13]. Yet, a sizable number of human cancers are also FDG-negative and a gradation of glycolic indices is reported within many tumor types that may or may not reflect specific lesions. Some positive regulators of cell proliferation are associated with reduced hexokinase activities and diminished glycolysis. For instance, antisense-mediated cyclin D1 down-modulation has been shown to upregulate glycolysis in vivo, by releasing hexokinase II from inhibition [38]. These contradictions perhaps reflect the intricacies underlying the cancer-associated metabolic networks and their confounding heterogeneity. In medulloblastoma, a unique, albeit more defined, metabolic pattern controlled by Shh is beginning to emerge: The striking accumulation of lipid droplets we describe earlier ([2] and Fig. 1a), is matched by the equally striking glycolytic index and confirmed by elevated FDG-PET activity. This phenotype is consistent with substrate utilization patterns in highly anabolic tissues where excessive glucose uptake is routed to fat production and excessive lipogenesis. Moreover, this particular substrate utilization pattern in shh-driven MBs unravels a new circuitry coupling hedgehog signaling directly to glucose uptake. At the heart of this process is the Rb/E2F complex, itself the target of growth factors and external trophic signals and a tumor suppressor broadly inactivated in human cancers. Our work identified the (Shh ⊣ Rb ⊣ E2F1 → PPAR) axis as a functional metabolic driver of medulloblastoma tumor formation and the core of its nutrient sensing network coupling external dietary conditions to the metabolic adaptation of the cellular milieu, including the regulation of the glycolic index. We note that in CGNPs, the mere incubation of primary cells with Shh markedly triggers Glut4—a glucose transporter typically induced with insulin stimulation. As such, glucose utilization in neural precursors could be primarily a Shh-regulated event, irrespective of hormonal status. Indeed, our data implicate this E2F1-dependent glycolytic process as a systematic, distinctly cell-autonomous process that is mediated by PPARγ but triggered by Shh in medulloblastoma.

## Electronic supplementary material

Below is the link to the electronic supplementary material.

Supplementary material 1 (TIFF 15323 kb)

Supplementary material 2 (TIFF 12268 kb)

Supplementary material 3 (TIFF 12100 kb)

Supplementary material 4 (TIFF 9366 kb)
