# Supplementary figures and images for: Hedgehog-mediated regulation of PPARγ controls metabolic patterns in neural precursors and shh-driven medulloblastoma
Source: Acta Neuropathol. 2012 Mar 11;123(4):587–600. doi: 10.1007/s00401-012-0968-6 (PMC3306783; doi:10.1007/s00401-012-0968-6)

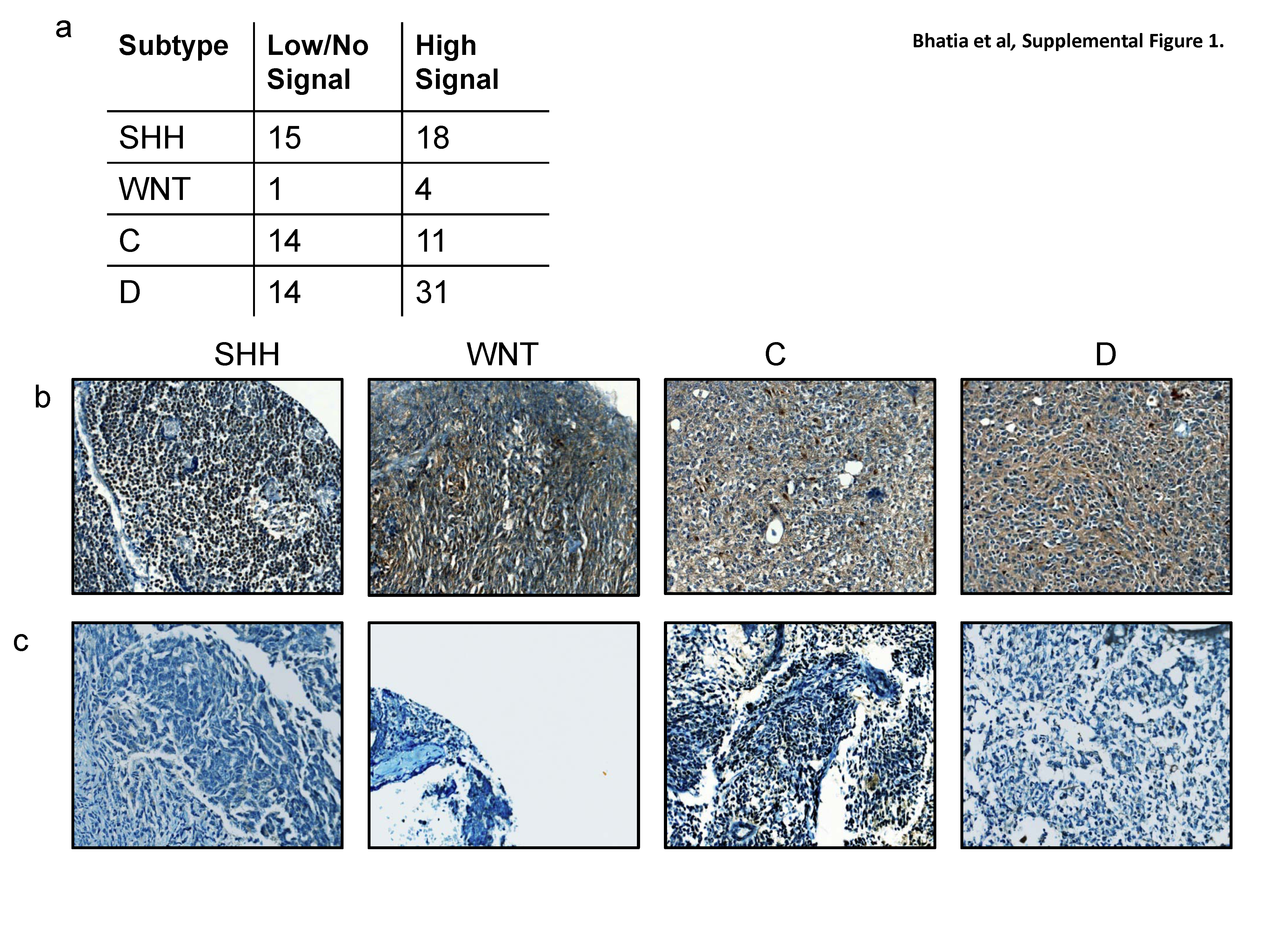

Supplement: Supplementary file 1 — Supplementary material 1 (TIFF 15323 kb) [file 401_2012_968_MOESM1_ESM.tiff]

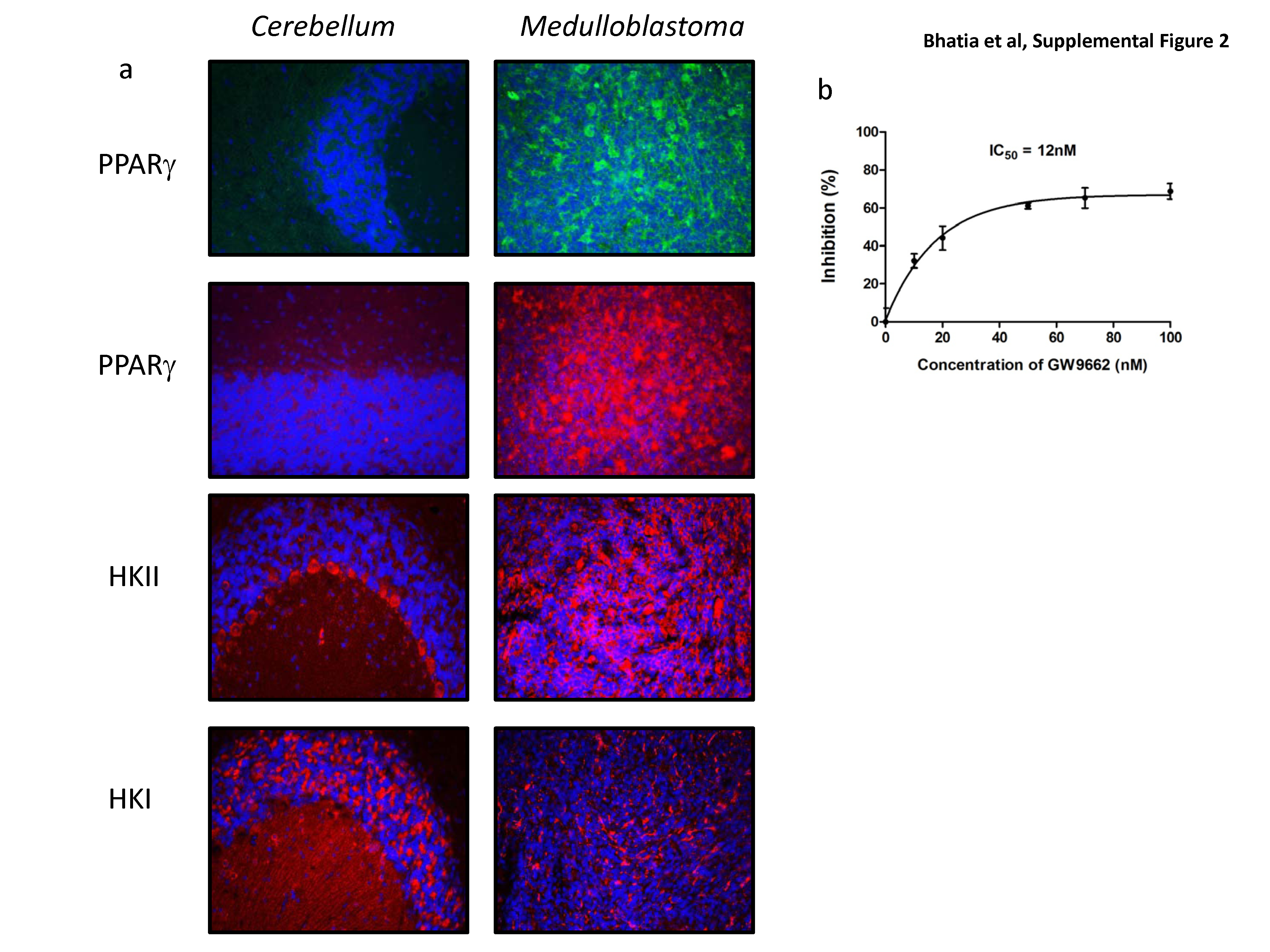

Supplement: Supplementary file 2 — Supplementary material 2 (TIFF 12268 kb) [file 401_2012_968_MOESM2_ESM.tiff]

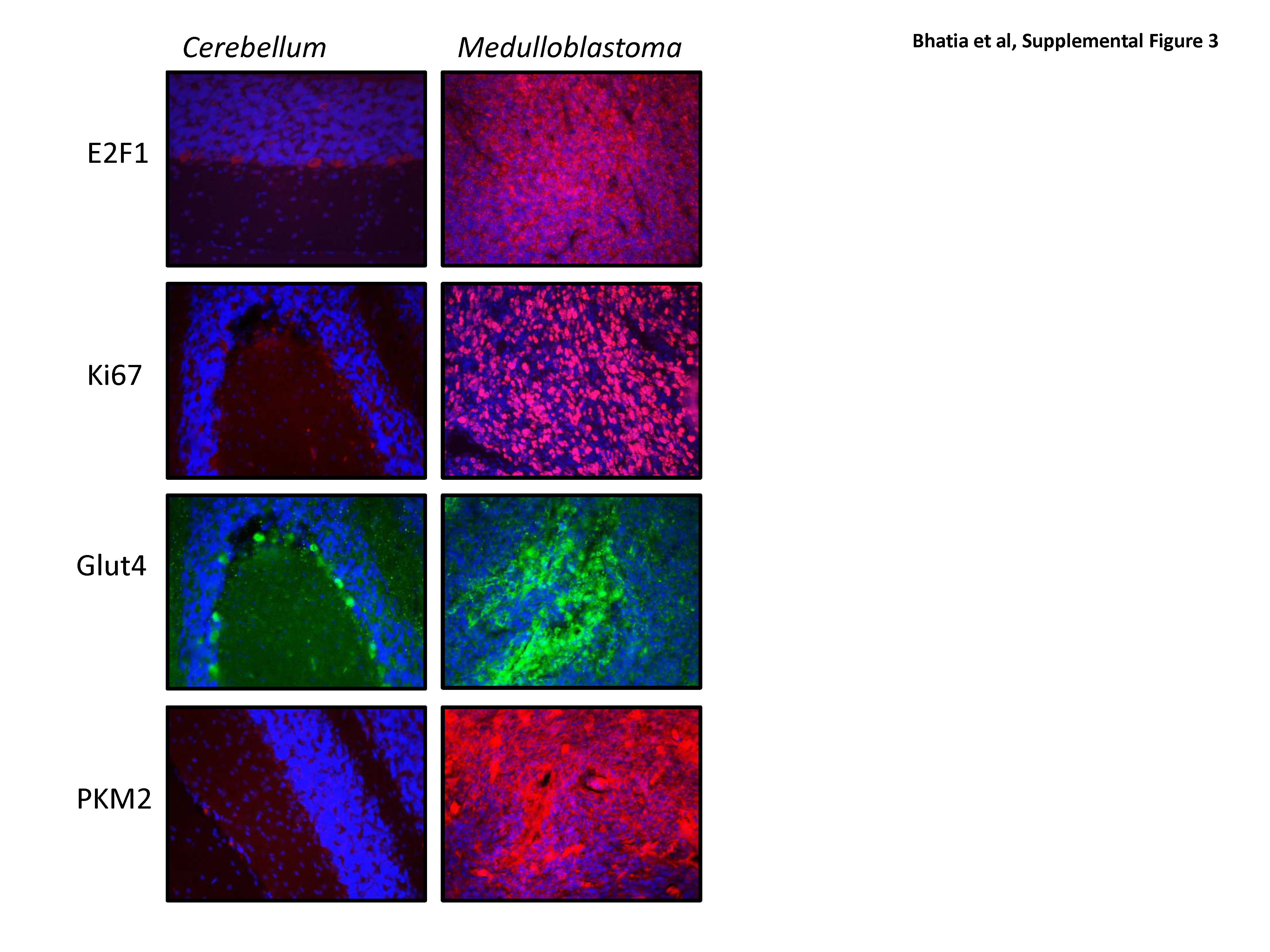

Supplement: Supplementary file 3 — Supplementary material 3 (TIFF 12100 kb) [file 401_2012_968_MOESM3_ESM.tiff]

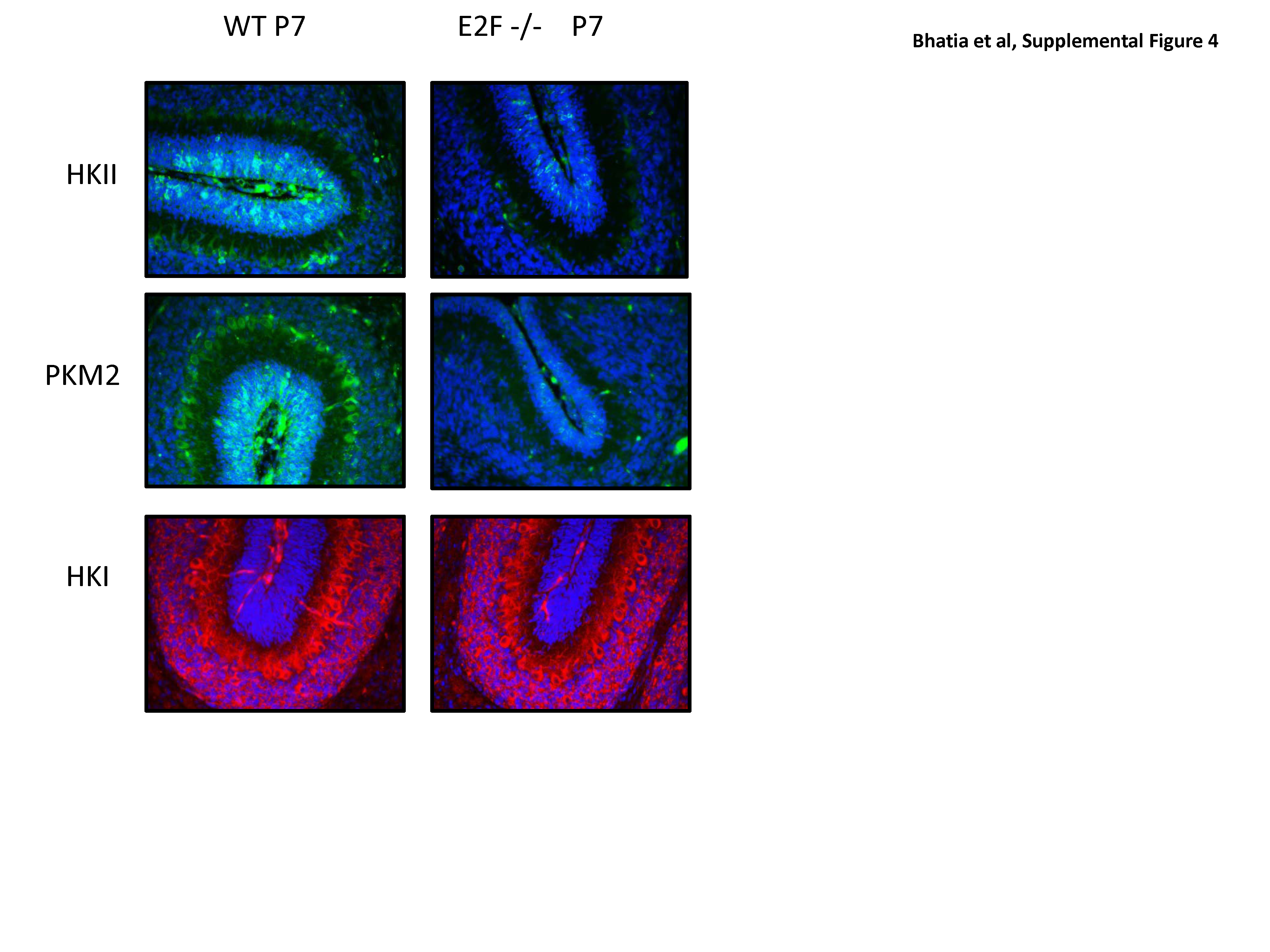

Supplement: Supplementary file 4 — Supplementary material 4 (TIFF 9366 kb) [file 401_2012_968_MOESM4_ESM.tiff]
